# Human T-lymphotropic virus-1 infection among Latin American pregnant women living in Spain

**DOI:** 10.1016/j.ijregi.2023.11.010

**Published:** 2023-11-22

**Authors:** Begoña Encinas, Rafael Benito, Silvia Rojo, Gabriel Reina, Natalia Montiel, Antonio Aguilera, José María Eiros, Juan García-Costa, Diego Ortega, Irene Arco, Araceli Hernánez-Batancor, Vicente Soriano, Carmen de Mendoza, Spanish HTLV Network

**Affiliations:** 1 Gynecology & Obstetrics Department, Puerta de Hierro University Hospital, Madrid, Spain; 2 Microbiology Department, Hospital Clínico Universitario Lozano Blesa, Zaragoza, Spain; 3 Microbiology Department, Hospital Clínico Universitario, Valladolid, Spain; 4 Microbiology Department, Clínica Universidad de Navarra, Pamplona, Spain; 5 Microbiology Department, Hospital Universitario Puerta del Mar, Cádiz, Spain; 6 Microbiology Department, University of Santiago, Santiago de Compostela, Spain; 7 Microbiology Department, Rio Hortega University Hospital, Valladolid, Spain; 8 Microbiology Department, Complejo Hospitalario de Ourense, Ourense, Spain; 9 Microbiology Department, Hospital Miguel Servet, Zaragoza, Spain; 10 Microbiology Department, General University Hospital of Alicante, Pintor Baeza, Alicante, Spain; 11 Microbioligy Department, Hospital insular Unidad De Traslados, Avenida Maritima del Sur, Las Palmas de Gran Canaria, Spain; 12 International University of La Rioja (UNIR) Health Sciences School & Medical Center, Madrid, Spain; 13 Puerta de Hierro University Hospital & Research Foundation-IDIPHISA, Madrid, Spain

**Keywords:** Pregnant women, Vertical transmission, HTLV-1, Breastfeeding, Migration

## Abstract

**Objectives:**

Human T-lymphotropic virus (HTLV) antenatal screening is not mandatory in Spain. Surveys conducted decades ago reported HTLV-1 seroprevalence rates of 0.2% among foreign pregnant women in Spain. The migrant flow to Spain from HTLV-1 endemic regions in Latin America and sub-Saharan Africa has increased during the last decade. Currently, 25% of pregnant women in Spain are foreigners.

**Methods:**

From January 2021 to October 2023 a cross-sectional study was carried out in all consecutive pregnant women attended at eleven Spanish clinics. A commercial enzyme immunoassay (EIA) was used for screening of serum HTLV-1/2 antibodies. Reactive samples were confirmed by immunoblot.

**Results:**

A total of 9813 pregnant women with a median age of 34 years-old were examined. Native Spaniards were 6977 (76.5%). Of 2147 foreigners (23.5%), 903566 (9.9%) were Latin Americans, 416 (4.5%) North Africans, 293 (3.2%) from Romania, and 196 (2.1%) from sub-Saharan Africa. A total of 47 samples were EIA reactive but only five were confirmed as HTLV-1 positive using immunoblot. Infected women came from Paraguay, Colombia, the Dominican Republic, Venezuela and Peru. All but one were primigravida, with ages ranging from 20 to 33 years-old. One was HIV-1 positive, and another was infected with *Chlamydia trachomatis*.

**Conclusion:**

The overall seroprevalence for HTLV-1 among pregnant women in Spain is 0.05% but rises ten-fold (0.55%) among Latin Americans. This rate is higher than in surveys conducted decades ago. Our results support that anti-HTLV testing should be part of antenatal screening in Spain in pregnant women coming from Latin America, as it is already done with Chagas disease.

## Highlights

• Human T-lymphotropic virus-1 (HTLV-1) antenatal testing is not recommended in most countries.
• Avoidance of breastfeeding largely prevents mother-to-child HTLV-1 transmission.
• A total of 7252 pregnant women were tested for HTLV antibodies in Spain.
• Four were HTLV-1+ (rate 0.06%). All were Latin Americans (rate in this group 0.7%).
• Antenatal HTLV testing should be performed along with Chagas disease screening.

## Introduction

Infection with human T-lymphotropic virus-1 (HTLV-1) is a neglected condition, despite being the second most prevalent human retroviral infection worldwide after HIV-1. Current estimates are of 10 million people living with HTLV-1 globally, with high endemic regions located in Equatorial Africa [1,2], Latin America and the Caribbean, northeastern Iran, southwestern Japan, and Australia [3], [4], [5].

The virus is transmitted mostly cell-to-cell following exposure to contaminated biological fluids through sexual contact [6,7]; vertically from mother-to-child and breastfeeding [8]; and parenterally, such as in injection drug users [9], recipients of blood transfusions, or solid organ transplants [10]. Once acquired, HTLV-1 infection is lifelong [11]. Although many HTLV-1-infected individuals are asymptomatic and unaware of their infection, roughly 10% will go to develop clinical manifestations, generally after several decades of silent infection, being the most characteristic conditions HTLV-1-associated myelopathy (HAM) [12] and adult T-cell leukemia/lymphoma (ATLL) [13].

The diagnosis of HTLV-1 in pregnant women is important for multiple reasons. First, because it will help effectively prevent HTLV-1 transmission to the newborn in most cases, just precluding extended breastfeeding [14,15]. Indeed, vertical contagion may go down from 20% to 2-5% if breastfeeding is avoided [16]. Second, unveiling the new HTLV-1 diagnosis in the mother will favor her clinical assessment and follow-up, making early diagnosis possible for potential future clinical complications, such as development of HAM or ATLL. Third, contact tracing would help identify other HTLV-1 individuals, such as sexual partners, parents, siblings, and other children of the newly diagnosed pregnant women. Fourth, it would help prevent further HTLV-1 infections, either sexually or vertically.

HTLV-1/2 antenatal screening is not mandatory in Spain, even for women coming from HTLV-1 endemic areas. Surveys conducted more than one decade ago reported HTLV-1 seroprevalence rates of 0.2% among foreign pregnant women in Spain [17], [18], [19]. Interestingly, the migrant flow to Spain from Latin American women of childbearing age has increased approximately 3-fold during the last decade compared to prior years [20]. Roughly 25% of current pregnant women in Spain are foreigners [20]. Herein, we report the results of a recent nationwide cross-sectional study of anti-HTLV antibodies in consecutive pregnant women in Spain.

## Methods

A nationwide HTLV-1 register was created in Spain in 1989. Main demographics, clinical symptoms/signs, and laboratory findings are collected for each new HTLV-1 case at baseline and longitudinally using a standardized case report form. Members of the Spanish HTLV Network cover nationwide most of the lab facilities where this virus can be diagnosed, including public or private microbiology labs or blood banks [21]. From the coordination center, all members of the network were invited to participate in the current study.

From January 2021 to October 2023 a cross-sectional study was carried out in all pregnant women attending eleven different hospitals distributed across the Spanish geography. A commercial enzyme immunoassay (EIA), Abbott Architect rHTLV I/II assay, was used as screening for serum HTLV-1/2 antibodies. Reactive samples were further confirmed by immunoblot (Inno-Lia HTLV-I/II). Demographics (age, country of origin) and other concomitant infections were recorded in all cases.

### Statistical analysis

Figures are given in absolute numbers and percentages. Quantitative and qualitative variables are described as medians with interquartile ranges, mean with standard deviations or as proportions. Bivariate comparisons of quantitative variables were performed using the Chi^2^ test.

All statistical analyses were performed using the IBM SPSS package for Windows v25.0 (IBM Corp, Armonk, NY). All tests were two-tailed and only *P*-values <0.05 were considered as significant.

### Ethical approval

The study was designed as a multicentre and retrospective collection of anonymized and consecutive clinical data associated with serum HTLV-1 antibodies. It was approved by the International University of La Rioja (UNIR) ethics committee (ref. PI047/2023) and Puerta de Hierro University Hospital ethics committee (ref. PI 10/20).

## Results

A total of 9813 consecutive pregnant women with a median age of 34 years were examined. Native Spaniards were 6977 (76.5%). Of 2147 foreigners (23.5%), 903 (9.9%) were Latin Americans, 416 (4.5%) North Africans, 293 (3.2%) from Romania, and 196 (2.1%) from sub-Saharan Africa. It was unknown for 689 (7%).

A total of 47 samples were initially reactive by EIA but only 5 were confirmed as HTLV-1 positive using immunoblot. None were positive for HTLV-2. Infected women came from Paraguay, Colombia, the Dominican Republic, Venezuela and Peru (Table 1).

**Table 1:** Main features of pregnant women in Spain infected with human T-lymphotropic virus-1.

Case	Age (years-old)	Country of origin	Year of diagnosis	City of residence	Other infections
1	31	Paraguay	2021	Madrid	No
2	23	Colombia	2022	Madrid	No
3	20	Dominican Republic	2023	Zaragoza	HIV-1
4	22	Peru	2023	Zaragoza	*Chlamydia trachomatis*
5	33	Venezuela	2023	Madrid	No

The five newly diagnosed HTLV-1 women were living in Madrid (2) and Zaragoza (2). One was HIV-1 positive, and another was infected with *Chlamydia trachomatis*. All but one were primigravida, with ages ranging from 20 to 33 years. The 31 year-old infected woman from Paraguay had already another child who had been breastfed years earlier. The boy is currently living in his country of birth and has not been tested yet for HTLV-1.

## Discussion

In our current study, we found an overall seroprevalence for HTLV-1 among pregnant women in Spain of 0.05%. It rose ten-fold (0.55%) among Latin Americans. These rates are higher than those seen in prior surveys conducted more than one and two decades ago in Spain [17], [18], [19].

In Spain, three nationwide studies have been performed previously examining the rate of HTLV infection among pregnant women. The oldest study was conducted in 20,366 pregnant women who attended clinics between 1996 and 1999. Two were positive for HTLV-1 (one from Peru) and 11 for HTLV-2. The overall seroprevalence for HTLV was 0.06% [17], as in the current study.

The second study of antenatal HTLV in Spain was conducted with 20,518 pregnant women who attended clinics in 2006 and 2007, of which 18,266 (89%) were native Spaniards [18]. A total of 946 (4.6%) were from HTLV endemic regions in Latin America and Africa. Two were positive for HTLV-1 and two for HTLV-2. Hence, the overall seroprevalence for HTLV was 0.02%. It rose to 0.2% among pregnant women coming from HTLV-1 endemic regions.

The third antenatal study conducted in Spain included 3,337 foreign pregnant women who attended clinics in 2009 and 2010. Overall, 47% were Latin Americans and 24% were Africans. A total of six HTLV-1 (five from Latin America and one from Africa) and one HTLV-2 (from Ghana) were identified. Hence, the overall HTLV seroprevalence was 0.2%, being 0.3% among Latin American pregnant women [19].

The largest collaborative European study that examined antenatal HTLV infection was conducted 20 years ago [22]. A total of 234,078 pregnant women who attended clinics at seven European countries were examined. Positivity for HTLV-1 was found in 73 and for HTLV-2 in 17. The overall HTLV seroprevalence was 0.04%, roughly 6-fold higher than in blood donors tested at that time.

Prevention of HTLV-1 mother-to-child transmission is considered a priority to tackle this lifelong infection, for which there is neither a curative treatment nor a vaccine [4]. Screening policies for neglected infections, such as HTLV-1, vary in distinct countries, and go from universal testing to targeting only high-risk populations, recommend testing just once in life, testing only sex partners and/or relatives, or request testing only when there is high clinical suspicion.

Universal HTLV-1 antenatal screening was implemented in Japan in 2010, and it was recommended nationally in Brazil in April 2022. These two countries are the ones with the largest number of HTLV-1 infected persons worldwide. Exclusive formula feeding is recommended to mothers with HTLV-1 infection. In Brazil, where the prevalence of HTLV-1 among pregnant women has been estimated around 0.32% [23], recent cost-effective analysis has demonstrated the convenience of introducing universal antenatal HTLV screening [24].

At the recent HTLV European Research Network (HERN) held in Madrid, Spain, UK researchers estimated that 74 HTLV-1 infected babies per year would be avoided in the UK following the introduction of universal screening of pregnant women. This figure takes into consideration the number of pregnant women at risk of infection mostly from Africa and the Caribbean living in the UK; and the 15% rate of vertical HTLV transmission when breastfeeding is not avoided [25]. Using a similar approach in Spain, we estimated that roughly 35 HTLV-1 infected babies should be avoided annually if HTLV antenatal screening became mandatory. Given that most pregnant women from Latin America living in Spain are already being tested for *Trypanosoma cruzii* (Chagas disease) [26], adding HTLV serological screening would not add a significant job load.

We should acknowledge several limitations of our study. The sample population was not representative for all the country and exclusive sites, as no pregnant women from the Canary Islands and the Balearic Islands were included in the study. It should be noticed that these sites might include a larger number of pregnant women coming from HTLV-1 endemic regions, since their migrant populations are greater than in mainland Spain. Another caveat of our study is that pregnancies were tested during the COVID-19 pandemic period, when its number significantly declined associated with social isolation procedures and global and individual's economic and anthropological uncertainty [27]. However, sampling started after the two major first COVID-19 waves, when fertility rates began to recover in most Western countries.

There are two caveats with respect to the interpretation of our results that would be worth to discuss. First, the specificity of the EIA screening assay was low: only 4 out of 44 initially seroreactive samples were confirmed as HTLV-1 positive using immunoblot. This poor specificity of the commercial assay during pregnancy already has already been highlighted by others [28] and could be associated with the more frequent occurrence of hypergammaglobulinemia in persons from tropical/subtropical regions and/or during gestation. Increasing the threshold for considering reactivity could ameliorate the high rate of false positives without compromising sensitivity [29]. This approach is worthy in the transplantation setting where organ/tissue wastage could be problematic. However, it is less relevant during pregnancy since confirmatory tests can be performed without such urgency.

Another caveat refers to recommend testing to all Latin American pregnant women, when high HTLV-1 endemicity has been demonstrated only for some but not all countries in the continent. Even within the same country, hot spots are recognized whereas other regions depict low HTLV-1 rates [23]. In Spain, the largest migration flow from Latin America includes most countries with confirmed high HTLV-1 endemicity such as Brazil, Colombia, Venezuela, Peru, Ecuador, Bolivia, Dominican Republic, Argentina, Chile, etc. [3]. On top of that, the Spanish HTLV register has alerted about a progressive erase of microbiological edges between countries in Latin America due to increasing migration flows within the region [21].

## Conclusion

In this cross-sectional nationwide study, we found a current overall rate of HTLV-1 infection of 0.05% among pregnant women in Spain that increased ten-fold (up to 0.55%) among Latin Americans. Hence, our results support that anti-HTLV testing should be part of antenatal screening in Spain in all pregnant women coming from Latin America, as it is already being done with Chagas disease [26].

## Declarations of Competing Interest

The authors have completed the information regarding conflicts of interest in the requested documentation and have no conflicts of interest to disclose.
